# Low-Terahertz Transparent Graphene-Based Absorber

**DOI:** 10.3390/nano10050843

**Published:** 2020-04-28

**Authors:** Alessandro Giuseppe D’Aloia, Marcello D’Amore, Maria Sabrina Sarto

**Affiliations:** 1 Department of Astronautical, Electrical and Energy Engineering, Sapienza University of Rome, 00184 Rome, Italy; marcello.damore@uniroma1.it (M.D.); mariasabrina.sarto@uniroma1.it (M.S.S.); 2 Research Center for Nanotechnology applied to Engineering of Sapienza University, 00185 Rome, Italy

**Keywords:** low-terahertz transparent absorber, graphene-polyethylene terephthalate laminate, laminate effective medium model, transmission line approach, absorption, reflection and transmission performance up to 4 THz, optical transmittance in the visible range

## Abstract

A new, transparent, metal-free absorber, based on the use of multilayer graphene/dielectric laminates (GLs), is proposed for applications in the low-terahertz frequency range. The designed absorber has a total thickness of around 70 µm and consists of a front matching dielectric layer followed by a GL, a dielectric spacer and a back GL. The laminates are periodic structures constituted of graphene sheets separated by 50-nm-thick polyethylene terephthalate (PET) interlayers, while the matching layer and the spacer are one-quarter-wavelength thick and made of PET. The GLs are modeled as homogeneous-equivalent single layers (ESLs) characterized by their sheet resistances *R_s_*. An innovative analytical method is proposed in order to select *R_s_* values optimizing the electromagnetic wave absorption either in low-gigahertz or low-terahertz frequency range. The frequency spectra of the absorption, reflection and transmission coefficients are computed in the range up to 4 THz by using different values of *R_s_*. Then, realistic *R_s_* values of chemically doped graphene monolayers over PET substrates are considered. The designed absorbers are characterized by an absorption coefficient with a peak value of about 0.8 at the first resonant frequency of 1.1 THz, and a 1.4 THz bandwidth centered at 1.5 THz with reflection coefficient below - 10 dB. Moreover, the optical transmittance of the proposed absorbers are computed by means of the optical matrix theory and it is found to be greater than 86% in all the visible ranges.

## 1. Introduction

In the last decade, the exponential growth of wireless communications and data traffic has fueled the research in technologies operating at terahertz (THz) and low-THz frequency ranges [1]. Moreover, these technologies are attracting ever-growing interest in many other industrial and research fields, such as biomedical imaging and signal detection, as well as stealth materials and sensing [2]. Therefore, intensive efforts have been made to fabricate novel, transparent and multi-functional devices operating at such frequency regimes. In particular, the development of new absorbers operating in the low-THz and THz frequency range is generally recognized as an urgent challenge to be addressed, since high absorption is crucial for several applications such as modulators, sensors and solar cells [3,4]. Recently, resonance-based metamaterial absorbers have aroused intensive interest [5,6], since they can offer near unity absorption. However, the complexity of these structures leads to difficulties in fabrication because most of them employ metals, classified as critical raw materials and characterized by electrical and electromagnetic properties hardly tunable, especially when thin films are required [4,7]. Furthermore, most of these structures are not transparent in the visible frequency range, reducing their applications.

In this context, graphene, which is a two-dimensional honeycomb structure of carbon atoms, has gained a lot of interest due to its exceptional electrical, thermal, mechanical and optical properties [8]. Currently, two-dimensional graphene layers are characterized by optical transmittance greater than 90% (as a comparison, the Indium tin oxide transmittance is around 81%) and their conductivity can be tuned through electrical or chemical doping.

For instance, single-frequency, dual-frequency and wide-band absorbers made of graphene-based metasurfaces and characterized by large angle tolerance properties in the THz spectrum up to 4 THz are proposed in [3]. A tunable THz absorber employing a 5-nm-thick interlayer of Al_2_O_3_ is presented in [9] and an accurate procedure for the design of multilayer THz absorbers employing tunable graphene metamaterials has been developed in [10], with the aim of obtaining high absorption over a wide-THz frequency band. Moreover, in [11] tunable double-layer graphene wires are combined with metamaterials having unit cells of cross-shaped metallic resonator in order to realize a polarization-independent absorber with spectral tuning at THz frequency. Then, a hyperbolic metamaterial consisting of a multilayer structure of alternating graphene and Al_2_O_3_ layers on a CaF2 substrate is realized and characterized in [12]. More recently, a tunable ultra-broadband THz absorber based on two layers of graphene ribbons and two different kinds of substrate materials is proposed in [13] while an extensive study on broadband near-perfect absorbers consisting of single-layered and non-structured graphene loaded with periodical arrays of dielectric bricks is presented in [14]. Finally, in [15] a reliable method to extract the electromagnetic constitutive tensors of complex synthesized multilayer graphene-dielectric stacks in the terahertz band is derived.

Nevertheless, the realization of periodic graphene nanostructures is still not an easy task. Recently, Salisbury absorber screens, consisting of a thin resistive film, a dielectric spacer and a backside reflector, have attracted growing interest for the realization of graphene-based THz absorbers [7,13,14,15,16,17,18]. In fact, graphene films can act as resistive films [7] and, in addition, their conductivity can be tuned in order to obtain optimum absorption. For instance, numerical studies show that graphene ribbon arrays coupled with a graphene sheet can achieve a near unity absorption in a wide-THz bandwidth by changing graphene electrical properties through electrical doping due to an applied voltage [14,16]. A similar electrical doping has been investigated in [4,17]. Moreover, a new adaptive Salisbury absorbing screen providing a reflection coefficient lower than -10 dB in the frequency range 0.6–2.2 THz and with total thickness around 70 μm is proposed in [18]. The designed screen, backed by a perfect electrical conducting plate, consists of firstly a graphene layer acting as a lossy sheet and two spacers made of polyethylene terephthalate (PET), separated by an electrically tunable graphene/dielectric laminate (GL). Numerical analyses have been performed on the basis of the equivalent models and the results described in [19,20,21,22].

The objective of this paper is the design of new two-periods transparent metal-free absorbers made of multilayer graphene-PET laminates. The materials are chosen in order to obtain an optically transparent screen, and to avoid the use of metals or other critical raw materials, also for the backside reflector, which is usually made of a thin gold layer. The GLs are modelled as homogeneous-equivalent single layers (ESLs) characterized by proper sheet resistances, whose values are obtained analytically in order to achieve good absorption performances. The developed analytical method is innovative since it provides a simple and immediate tool that can be applied to design electromagnetic absorbers in the low-THz frequency range. Finally, the optical transmittances of the backside reflector and of the overall proposed absorbers are computed by means of the well-known matrix theory for the analysis of multilayer systems [23]. It results that the optical transmittance of the designed absorber is greater than 88% in all the visible ranges.

## 2. Absorber Structure

**Figure 1:**
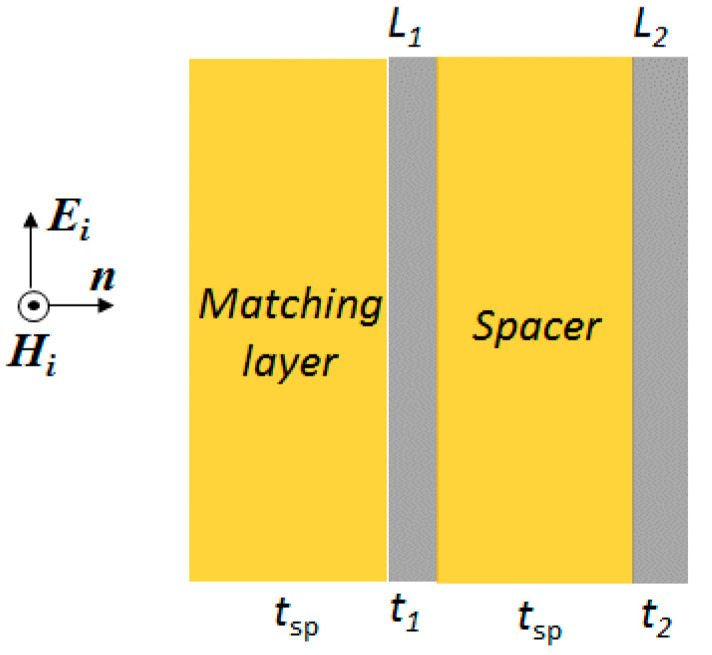
Schematic structure of the cross-section of the planar absorber illuminated by a plane wave.

The schematic structure of the proposed graphene-based absorber is represented in Figure 1. The absorber consists of a front matching PET layer followed by a graphene/PET laminate *L*_1_, a PET spacer and a back graphene/PET laminate *L*_2_. The matching layer and the spacer have a one-quarter-wavelength thickness *t*_sp_; *t*_1_ and *t*_2_ are the thicknesses of the laminates *L*_1_ and *L*_2_, respectively.

The absorber is assumed to have infinite transverse dimensions, and to be illuminated by a plane wave with normal incidence.

PET is selected for the realization of the spacer and matching layer due to its mechanical properties, optical transparency and cost-effectiveness. Moreover, graphene films over PET substrates are nowadays commercially available and they are characterized by outstanding electrical and mechanical properties [24]. Another polymer commonly used as substrate for graphene is polymethylmethacrylate (PMMA). However, PMMA films are characterized by worse mechanical properties than PET ones.

**Figure 2:**
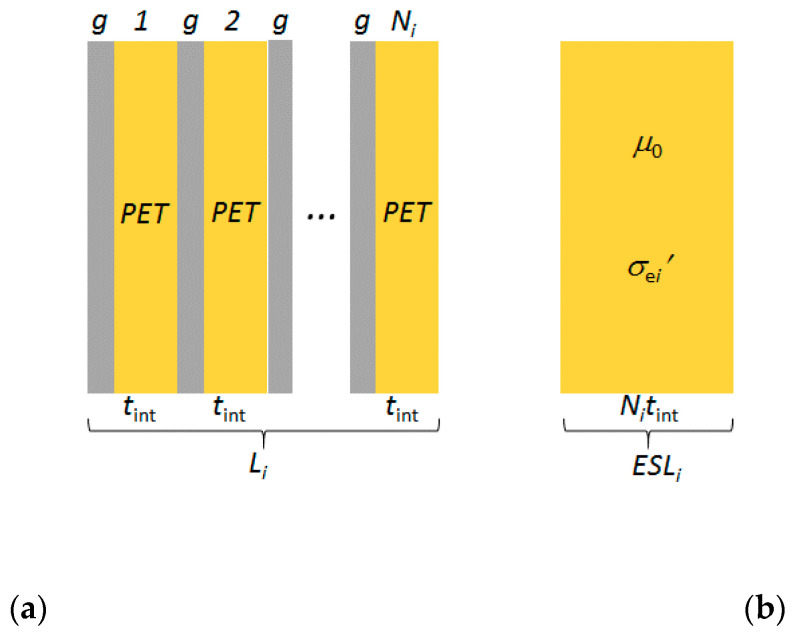
Schematic structure of (**a**) graphene-polyethylene terephthalate (PET) laminate *L_i_* and (**b**) its equivalent single layer (ESL*_i_*).

The *i*th laminate *L_i_* of Figure 2a consists of a periodic structure constituted of *N_i_* bilayers made of a graphene sheet separated by a PET interlayer having thickness *t_i_*_nt_. Thus, the thickness of each graphene-PET bilayer is *t*_b_ = *δ* + *t_i_*_nt_, *δ* being the graphene thickness, conventionally assumed equal to 0.34 nm. It results *t*_b_ ≅ *t_i_*_nt_ and the total thickness of the *i*th laminate, *t_i_*, is equal to *N_i_t_i_*_nt_.

## 3. Graphene and Polymer Modeling

### 3.1. Graphene Conductivity

The frequency-dependent conductivity *σ*(*ω*) of an isolated graphene sheet can be represented by Kubo’s formalism, which takes into account the intraband and the interband contributions [25].

In the low-THz frequency band only the intraband conductivity term is considered, since the interband one is negligible. In fact it results $\hbar \omega <<2\;\left|\mu_{c}\right|$, i.e., $f<<f_{c}\approx 0.484\;\times 10^{15}\;\left|\mu_{c}\right|$, where *μ_c_* is the chemical potential in eV, ℏ=h/2π the reduced Planck’s constant (with *h* = 6.6262 × 10^−34^ Js) and *ω* the angular frequency. It yields [25]:

(1) $$\sigma \left(\omega \right)=-j\frac{e^{2}k_{B}T}{\pi \hbar^{2}\left(\omega -j/\tau \right)}\left[\frac{\mu_{c}}{k_{B}T}+2\ln\left(e^{-\mu_{c}/\left(k_{B}T\right)}+1\right)\right]$$

in which *e* = 1.602 × 10^−19^ C is the elementary electron charge, *k_B_* = 1.38064 × 10^−23^ J/K is the Boltzmann’s constant, *T* is the temperature in Kelvin and *τ* is the relaxation time of graphene.

It is worth noticing that if $\left|\mu_{c}\right|>>k_{B}T$, i.e., if $\left|\mu_{c}\right|>>0.0259\;\mathrm{eV}$ at *T* = 300 K, the exponential term in (1) becomes negligible and the intraband conductivity can be approximated by the following semi-classical Drude model:

(2) $$\sigma \left(\omega \right)=\frac{\sigma_{}}{1+j\omega \tau }$$

where *σ* is the dc conductivity:

(3) $$\sigma_{}=\frac{e^{2}\mu_{c}\tau }{\pi \hbar^{2}}$$

The scattering time can be expressed as a function of *μ_c_* and of the carrier mobility *μ* in the following form [22]:

(4) $$\tau =\frac{\mu \mu_{c}}{ev_{F}^{2}}$$

in which *v_F_* = 10^6^ m/s is the Fermi velocity. Therefore, Equation (3) can be rewritten as:

(5) $$\sigma =\frac{e\mu \;\mu_{c}^{2}}{\pi \;\hbar^{2}v_{F}^{2}}$$

The graphene dc conductivity *σ* at zero-field and zero-charge density is estimated to be close to the conductivity quantum 0.38 × 10^−4^ S and can reach the value of a few mS by increasing the chemical potential *μ_c_* through chemical or electrical doping [24].

The graphene layers composing the GL are assumed to be decoupled electronically thanks to the presence of dielectric interlayers with a thickness in the order of some tens of nanometers. Moreover, due to the infinite planar dimensions of the homogeneous graphene monolayers, plasmonic surface waves are neglected [26,27].

### 3.2. Polymer Permittivity

The PET-frequency-dependent relative permittivity can be predicted by using the Havriliak–Negami model [28]:

(6) $$\varepsilon_{r}\left(\omega \right)=\varepsilon_{r\infty }+\frac{\Delta \varepsilon_{R}}{{\left[1+{\left(j\omega \tau_{R}\right)}^{\;\beta }\right]}^{\;\alpha }}$$

where $\varepsilon_{r\infty }$ is the high-frequency value, $\Delta \varepsilon_{R}$ the relaxation strength, $\tau_{R}$ the relaxation time and *α* and *β* fitting parameters. According to [29], the best fit with experimental data is obtained when $\varepsilon_{r\infty }$ = 2.54, $\Delta \varepsilon_{R}$ = 0.46, $\tau_{R}$ = 0.05 ps, *α* = 2.9 and *β* = 0.9.

Thus, in the low-THz frequency range, the PET permittivity $\varepsilon_{r}\left(\omega \right)$ is a complex number, with a real part $\varepsilon_{rR}\left(\omega \right)$ and an imaginary part $\varepsilon_{rI}\left(\omega \right)$, that are frequency-dependent quantities. It yields:

(7) $$\varepsilon_{r}\left(\omega \right)=\varepsilon_{rR}^{}\left(\omega \right)+j\varepsilon_{rI}^{}\left(\omega \right)$$

**Figure 3:**
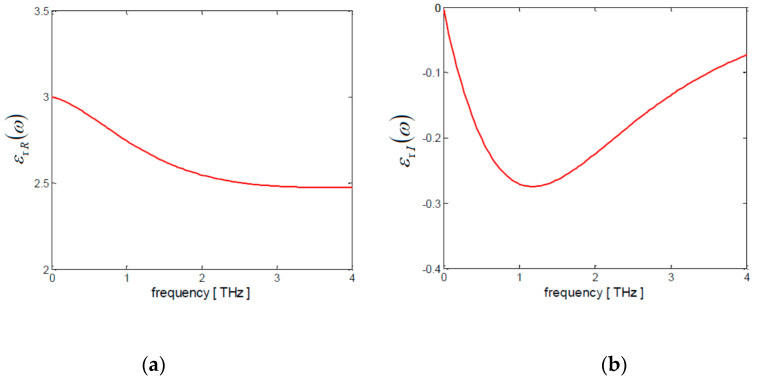
Frequency spectra of the (**a**) real and (**b**) imaginary part of the relative permittivity of PET polymer substrate.

The frequency spectra of $\varepsilon_{rR}\left(\omega \right)$ and $\varepsilon_{rI}\left(\omega \right)$ are shown in Figure 3a,b in the range from 0.01 THz up to 4 THz.

### 3.3. Optical Transmittance of Graphene and PET Laminate

According to [30,31], the optical transmittance *T*_opt_ _λ = 550 nm_ at the light wavelength *λ* equal to 550 nm of stacked graphene monolayers is given by:

(8) $$T_{\mathrm{opt}\;\lambda =550\mathrm{nm}}={\left(1+1.13N\pi \gamma /2\right)}^{-2}$$

where *N* is number of suspended graphene layers and *γ* is the fine structure constant, equal to 1/137 [32]. Thus, for a single layer the optical transmittance is 97%, whereas for *N* = 4 or *N* = 5 it is 90% or 88%. However, such transmittance values do not consider the substrate effects that may not be neglected in case of graphene-based structures, like the ones sketched in Figure 1 and Figure 2a.

Indeed, the optical transmittance of GLs can be computed through the standard thin-film optics approach, based on the Fresnel equations [23]. For such calculations, a complex refractive index ${\tilde{n}}_{g}$ is typically assumed for graphene [33], with real part $n_{g}$ corresponding to the graphene refractive index and imaginary part $k_{g}$ representing the graphene extinction coefficient. It yields:

(9) $${\tilde{n}}_{g}=n_{g}-jk_{g}$$

Very recent experiments have shown that in the visible range, $n_{g}$ is constant and equal to 3, while the graphene extinction coefficient is given by:

(10) $$k_{g}=\frac{\zeta }{3}Ĉ$$

with *ζ* being a constant equal to 5.446 μm^−1^ and *λ* the incident light wavelength expressed in micrometers [34].

Concerning the dielectric layers, for a typical PET sample the complex refractive index ${\tilde{n}}_{PET}$ is equal to the refractive index $n_{PET}$ (a real number), since the PET extinction coefficient $k_{PET}$ is zero in the visible range [35].

**Figure 4:**
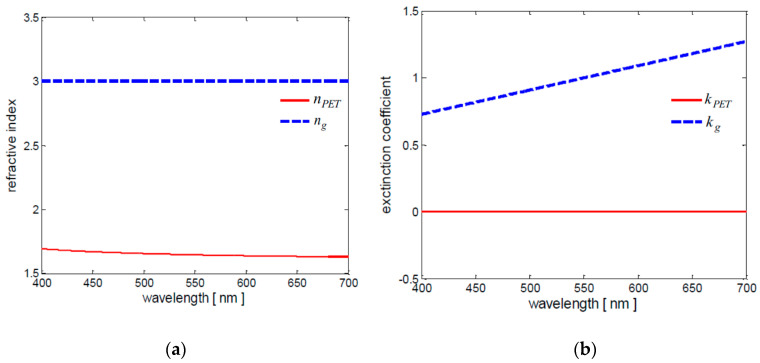
Refractive index (**a**) and extinction coefficient (**b**) of graphene (- - -) and PET (―) in the visible range.

The graphene and PET refractive indices and extinction coefficients are reported in Figure 4a,b, respectively, for wavelengths ranging between 400 nm and 700 nm.

## 4. Transmission Wave Modeling

### 4.1. Multilayer Model

The overall transfer matrix [Φ] of the absorber structure sketched in Figure 1 is [19]:

(11) $$\left[\Phi \right]=\left[\Phi_{\mathrm{sp}}\right]\;\left[\Phi_{L1}\right]\;\left[\Phi_{\mathrm{sp}}\right]\;\left[\Phi_{L2}\right]$$

in which [Φ*_Li_*] and [Φ_sp_] are the transfer matrices of the *i*th laminate *L_i_* and of the PET layers (either the spacer or the front matching layer).

The transfer matrix [Φ*_Li_*] is given by:

(12) $$\left[\Phi_{Li}\right]=\prod_{}^{N_{i}}\left[\Phi_{gi}\right]\left[\Phi_{\mathrm{int}}\right]$$

where [Φ_g*i*_] is the short-line transfer matrix of the *i*th single graphene layer with conductivity *σ_i_*(*ω*):

(13) $$\left[\Phi_{gi}\right]=\left[\begin{array}{cc} 1 & 0\\ \sigma_{i}\left(\omega \right) & 1 \end{array}\right]$$

and [Φ_int_] is the transfer matrix of the PET dielectric interlayer:

(14) $$\left[\Phi_{\mathrm{int}}\right]=\left[\begin{array}{cc} \cos\alpha_{\mathrm{int}} & j\eta_{s}\sin\alpha_{\mathrm{int}}\\ j\sin\alpha_{\mathrm{int}}/\eta_{s} & \cos\alpha_{\mathrm{int}} \end{array}\right]$$

The attenuation constant *α*_int_, the wavelength *λ_S_* and the characteristic impedance *η_S_* of the PET layers are respectively:

(15) $$\alpha_{\mathrm{int}}=2\pi t_{\mathrm{int}}/\lambda_{S}$$

(16) $$\lambda_{S}=\frac{c_{0}\sqrt{2}}{f\sqrt{\varepsilon_{rR}\left(\omega \right)+\left|\varepsilon_{r}\left(\omega \right)\right|}}$$

(17) $$\eta_{S}=\frac{\eta_{0}}{f\sqrt{\varepsilon_{rR}\left(\omega \right)+j\varepsilon_{rI}\left(\omega \right)}}$$

The transfer matrix [Φ_sp_] of the PET spacer and matching layer is given by Equation (14) replacing *α*_int_ with *α*_sp_ = 2 π*t*_sp_/*λ_S_*.

### 4.2. Equivalent Single-Layer Model of the Graphene-PET Laminate

In the low-THz frequency range, the PET interlayers with *t*_int_ = 50 nm behave as short lines (SLs), since it results (*t*_int_/*λ_s_*) << 1. Thus, the condition required to apply the homogenization procedure to the periodic structure given by the graphene-PET laminate of Figure 2a is satisfied [36]. Consequently, each multilayer laminate *L_i_* can be represented by the corresponding ESL*_i_* having thickness *t_i_* = *N_i_t*_int_, as shown in Figure 2b. The homogeneous ESL*_i_* is characterized by permeability *μ*_0_ and effective conductivity ${\sigma^{\prime }}_{ei}\left(\omega \right)$ expressed in s/m [36], given by:

(18) $${\sigma^{\prime }}_{ei}\left(\omega \right)=\frac{\sigma_{i}\left(\omega \right)}{t_{\mathrm{int}}}+j\omega \varepsilon_{0}\varepsilon_{r}\left(\omega \right)$$

that can be rewritten as:

(19) $${\sigma^{\prime }}_{ei}\left(\omega \right)={\sigma^{\prime }}_{eiR}\left(\omega \right)+j{\sigma^{\prime }}_{eiI}\left(\omega \right)$$

where the real and imaginary components, respectively ${\sigma^{\prime }}_{eiR}\left(\omega \right)$ and ${\sigma^{\prime }}_{eiI}\left(\omega \right)$, are:

(20) $${\sigma^{\prime }}_{eiR}\left(\omega \right)=\frac{\sigma_{i}}{t_{\mathrm{int}}\left[1+{\left(\omega \tau \right)}^{2}\right]}-\omega \varepsilon_{0}\varepsilon_{rI}\left(\omega \right)$$

(21) $${\sigma^{\prime }}_{eiI}\left(\omega \right)=\omega \left[\varepsilon_{0}\varepsilon_{rR}\left(\omega \right)-\frac{\sigma_{i}\tau }{t_{\mathrm{int}}\left[1+{\left(\omega \tau \right)}^{2}\right]}\right]$$

The transfer matrix [$\Phi_{\mathrm{ESL}i}^{}$] of the homogeneous ESL*_i_* is:

(22) $$\left[\Phi_{\mathrm{ESL}i}\right]=\left[\begin{array}{cc} \cosh\left(m_{i}t_{i}\right) & \eta_{i}\sinh\left(m_{i}t_{i}\right)\\ \sinh\left(m_{i}t_{i}\right)/\eta_{i} & \cosh\left(m_{i}t_{i}\right) \end{array}\right]$$

where *m_i_* and *η_i_* are the ESL*i* propagation constant and wave impedance, respectively given by:

(23) $$m_{i}=\sqrt{j\omega \mu_{0}{\sigma^{\prime }}_{ei}\left(\omega \right)}$$

(24) $$\eta_{i}=\sqrt{j\omega \mu_{0}/{\sigma^{\prime }}_{ei}\left(\omega \right)}$$

Note that the ESL*_i_* behaves as an SL since (*t_i_*/*λ*_ESL_*i*) << 0.1 in the considered frequency range. The wavelength *λ*_ESL*i*_ assumes the following new expression:

(25) $$\lambda_{\mathrm{ESL}i}=2\pi {\left\{\sqrt{\frac{\omega \mu_{0}}{2}\left[{\sigma^{\prime }}_{eiI}\left(\omega \right)+\left|{\sigma^{\prime }}_{ei}\left(\omega \right)\right|\right]}\right\}}^{-1}$$

Thus, in the case of SL hypothesis, the ESL*_i_* transfer matrix expressed in Equation (22) becomes:

(26) $${\left[\Phi_{\mathrm{ESL}i}\right]}_{SL}=\left[\begin{array}{cc} 1 & j\omega \mu_{0}t_{i}\\ \sigma_{ei}\left(\omega \right) & 1 \end{array}\right]$$

where *σ*_e*i*_(*ω*) is the equivalent effective conductivity, expressed in Siemens and computed as:

(27) $$\sigma_{ei}\left(\omega \right)=N_{i}t_{\mathrm{int}}{\sigma^{\prime }}_{ei}$$

Considering Equation (18), the equivalent effective conductivity can be rewritten as:

(28) $$\sigma_{ei}\left(\omega \right)=\frac{N_{i}\sigma_{i}}{1+j\omega \tau }+j\omega N_{i}t_{\mathrm{int}}\varepsilon_{0}\varepsilon_{r}\left(\omega \right)$$

### 4.3. Reflection, Transmission and Absorption Coefficients

The reflection *R* and transmission *T* coefficients of a multilayer absorber are given by the following expressions [18]:

(29) $$R=\left|\frac{1-Y_{in}\eta_{0}}{1+Y_{in}\eta_{0}}\right|$$

(30) $$T=2{\left|\Phi_{11}+\Phi_{22}+\eta_{0}\Phi_{21}+\Phi_{12}/\eta_{0}\right|}^{-1}$$

in which *Y_in_* is the absorber input admittance:

(31) $$Y_{in}=\frac{\eta_{0}\Phi_{21}+\Phi_{22}}{\eta_{0}\Phi_{11}+\Phi_{12}}$$

and Φ_11_, Φ_22_, Φ_12_, Φ_21_ are the coefficients of the absorber overall transfer matrix (11). If the SL hypothesis (*t_i_*/*λ*_ESL*i*_ ) << 0.1 is satisfied, the overall transfer matrix [Φ] can be computed using matrices [Φ_ESL*i*_]_SL_ instead of matrices [Φ_L*i*_] without losing accuracy.

Finally, the reflection coefficient in decibel *R_dB_* and the absorption coefficient *A* are obtained from *R* and *T* as:

(32) $$R_{dB}=20\log\left(R\right)$$

(33) A=1−T−R

## 5. Sheet Resistances of Graphene-PET Laminates

The absorption performances of the proposed transparent screens depend strongly on the laminate-sheet resistances that are functions of the number *N_i_* and dc conductivity *σ_i_* of the graphene layers. In order to obtain the optimum values of the sheet resistances, a new analytical formulation is developed on the basis of previously obtained results [37].

Each laminate *L_i_* is modeled as the corresponding homogeneous ESL*_i_* represented by the SL transfer matrix [ Φ_ESL*i*_]_SL_ expressed in Equation (26), in which the off-diagonal coefficients are $j\omega \mu_{0}t_{i}$ and $\sigma_{ei}(\omega )$. Assuming the low-frequency hypothesis, the term $j\omega \mu_{0}t_{i}$ can be neglected, being the total thickness of the ESL*_i_* in nanometric scale. Moreover, the equivalent effective conductivity $\sigma_{ei}(\omega )$ in Equation (28) can be approximated with the term *N_i_σ_i_*. Therefore, the transfer matrix [ Φ_ESL*i*_]_SL_ assumes the following simplified form.

(34) $${\left[\Phi_{\mathrm{ESL}i}\right]}_{SL}=\left[\begin{array}{cc} 1 & 0\\ G_{si} & 1 \end{array}\right]$$

where the sheet conductance *G_si_* can be written as:

(35) $$G_{si}=N_{i}\sigma_{i}$$

The thickness of the spacer and matching layer sketched in Figure 1 is assumed equal to *t*_sp_ = λ*_s_*_r_/4 in which $\lambda_{sr}=c_{0}/f_{r}\sqrt{\varepsilon_{r}}$ is the wavelength at the resonant frequency *f*_r_, being $\varepsilon_{r}=\varepsilon_{r\infty }+\Delta \varepsilon_{R}$, the low-frequency PET permittivity. Therefore, the transfer matrix [Φ_sp_]_r_ at the resonance frequency assumes the following form:

(36) $${\left[\Phi_{\mathrm{sp}}\right]}_{r}=\left[\begin{array}{cc} 0 & j\eta_{s}\\ j/\eta_{s} & 0 \end{array}\right]$$

in which $\eta_{s}=\eta_{0}/\sqrt{\varepsilon_{r}}$. Thus, the overall transfer matrix at the resonant frequency [Φ]_r_ is computed as:

(37) $${\left[\Phi \right]}_{r}=\left[\begin{array}{cc} 0 & j\eta_{s}\\ j/\eta_{s} & 0 \end{array}\right]\;\left[\begin{array}{cc} 1 & 0\\ G_{s1} & 1 \end{array}\right]\;\left[\begin{array}{cc} 0 & j\eta_{s}\\ j/\eta_{s} & 0 \end{array}\right]\;\left[\begin{array}{cc} 1 & 0\\ G_{s2} & 1 \end{array}\right]$$

The coefficients of matrix (37) are used to evaluate the corresponding input admittance *Y_in_*_r_ and the transmission coefficient *T*_r_. These quantities are expressed as functions of the sheet conductances *G_s_*_1_ and *G_s_*_2_. It yields:

(38) $$Y_{inr}=\left[G_{s2}+1/\eta_{0}\right]\times {\left[1+G_{s1}G_{s2}\eta_{0}{}^{2}/\varepsilon_{r}+G_{s1}\eta_{0}/\varepsilon_{r}\right]}^{-1}$$

(39) $$T_{r}=2{\left[2+\eta_{0}G_{s2}+\left(G_{s1}\eta_{0}{}^{2}/\varepsilon_{r}\right)\left(G_{s2}+1/\eta_{0}\right)\right]}^{-1}$$

Setting *Y_in_*_r_ and *T*_r_ respectively equal to 1/*η*_0_ and T*, where T* is a target value of the transmission coefficient at frequency *f*_r_, it is possible to obtain the optimum laminate-sheet conductances. It yields:

(40) $$G_{s1}=\frac{\varepsilon_{r}}{\eta_{0}}\left(\frac{1-2T^{*}}{1-T^{*}}\right)$$

(41) $$G_{s2}=\frac{1}{\eta_{0}}\left(\frac{1-2T^{*}}{T^{*}}\right)$$

with T* < 0.5. Notice that the latter equations can be used also in the low-gigahertz frequency range, as discussed in [37].

Considering a perfect electric conductor (PEC) back plate (i.e., *G_s_*_2_ →∞) instead of a graphene-dielectric laminate, Equation (38) and Equation (39) become respectively:

(42) $$Y_{inr}=\varepsilon_{r}/\left(\eta_{0}{}^{2}G_{s1}\right)$$

(43) $$T_{r}=0$$

Moreover, setting *Y_in_*_r_ equal to 1/*η*_0_, the sheet conductance of the first laminate is obtained from Equation (42) as:

(44) $$G_{s1}=\varepsilon_{r}/\eta_{0}$$

With the spacer thickness *t*_sp_ being equal to *λ_s_*/4, the peak values of the absorption coefficient occur at the following resonant frequency:

(45) $$f_{r}=\frac{c_{0}}{4t_{\mathrm{sp}}\sqrt{\varepsilon_{r}}}$$

It should be noted that the low-frequency graphene conductivity and PET permittivity considered in the previous equations decrease in the low-THz frequency band. Thus, the values of sheet conductances given by Equations (40), (41) or (44) cannot be considered as the ones that optimize the absorption performances, but as initial reference values in the design process.

## 6. Optical Transmittance Modeling

The optical transmittance *T*_opt_ of a material is defined as the ratio between the light intensity transmitted by the material to that incident upon it. The most general approach to compute *T*_opt_ is based on a matrix formulation of the boundary conditions at the layer surfaces [23].

In case of a normally incident beam of light on a multilayer structure placed in air, the optical transmittance *T*_opt_ can be computed as [38]:

(46) $$T_{\mathrm{opt}}={\left|\frac{2n_{0}}{n_{0}B+C}\right|}^{2}$$

where *n*_0_ is the air effective refractive index, conventionally equal to 1, and *B* and *C* are the normalized total tangential electric and magnetic fields at the front interface of the absorbing structure sketched in Figure 1 [23]. According to the optical matrix formulation, *B* and *C* are given by:

(47) $$\left[\begin{array}{c} B\\ C \end{array}\right]=\left[M\right]\left[\begin{array}{c} 1\\ n_{0} \end{array}\right]$$

with [*M*] being the overall ray transfer matrix of the considered structure [23].

The ray transfer matrix [*M_Li_*] of the graphene laminate sketched in Figure 2a consisting of *N_i_* graphene sheets separated by *N_i_* PET interlayers is computed as:

(48) $$\left[M_{Li}\right]=\prod_{}^{N_{i}}\left(\left[M_{g}\right]\;\left[M_{\mathrm{int}}\right]\right)$$

where [*M_g_*] and [*M*_int_] are the ray transfer matrices of the graphene layer and of the PET interlayer. In particular, [*M_g_*] is

(49) $$\left[M_{g}\right]=\left[\begin{array}{cc} \cos\delta_{g} & j\sin\delta_{g}/{\tilde{n}}_{g}\\ j\sin\delta_{g}{\tilde{n}}_{g} & \cos\delta_{g} \end{array}\right]$$

With

(50) $$\delta_{g}=\frac{2\pi }{\lambda }\left({\tilde{n}}_{g}t_{g}\right)$$

being *t*_g_ and (${\tilde{n}}_{g}t_{g}$) the conventional and the effective optical thickness of a graphene layer. Then, matrix [*M*_int_] is obtained from Equations (49) and (50) replacing the graphene complex refractive index ${\tilde{n}}_{g}$ and thickness *t*_g_ with the PET complex refractive index ${\tilde{n}}_{PET}$ and thickness *t*_int_.

Finally, the overall ray transfer matrix [*M*] of the absorber structure sketched in Figure 1, is:

(51) $$\left[M\right]=\left[M_{sp}\right]\left[M_{L1}\right]\left[M_{sp}\right]\left[M_{L2}\right]$$

where matrix [*M*_sp_] of the PET spacer and matching layer is obtained from matrix [*M*_int_] replacing *t*_int_ with *t*_sp_.

## 7. Absorber Performance

The sheet conductances of the laminates *L*_1_ and *L*_2_ are computed by means of Equations (40) and (41) assuming *ε*_r_ = 3 and T^*^ equal to 0.1, 0.07 and 0.05. The obtained values of the corresponding sheet resistances *R_si_* = 1/*G_si_* are reported in Table 1.

**Figure 5:**
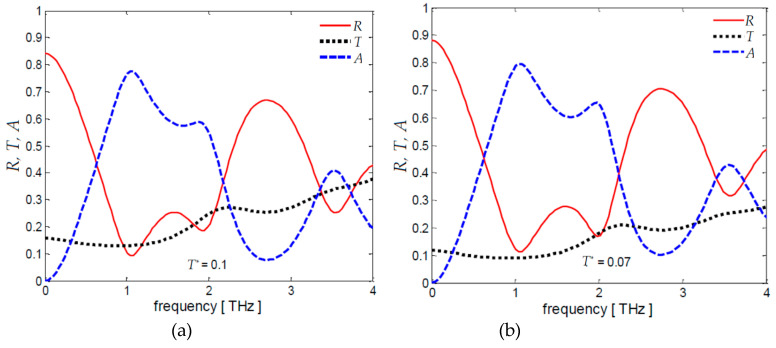

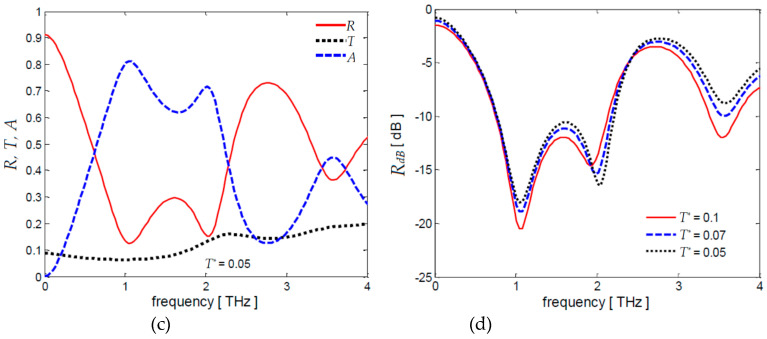
Frequency spectra of (**a**–**c**) coefficients *R, T, A* and (**d**) *R_dB_* for *τ* = 0.1 ps, *t_sp_* = 0.035 mm and different values of T^*^.

The reflection, transmission and absorption coefficients of the absorbers are computed in the frequency range 0.01–4 THz using Equations (29), (30), (32) and (33) combined with Equation (11). Each laminate *L_i_* is modeled through the ESL*_i_*-short-line transfer matrix of Equation (26) in which *σ*_e*i*_(*ω*) is given by Equation (28) where *N_i_σ_i_* is the sheet conductance *G*_s*i*_ of the *i*th laminate. The scattering time *τ* and the spacer thickness *t*_sp_ are assumed equal to 0.1 ps and 0.035 mm, respectively. The computed frequency spectra of *R, T, A* and *R_dB_* are represented in Figure 5 for different values of T^*^.

It is worth noticing that the absorption coefficient *A* increases slowly as T^*^ decreases and shows a peak value at 1.1 THz, i.e., the first resonant frequency, pretty close to 1.24 THz given by Equation (45) considering *ε*_r_ = 3. Furthermore, the transmission coefficient *T* at the resonant frequency assumes values close to the target values T^*^, i.e., 0.1, 0.07 and 0.05.

**Figure 6:**
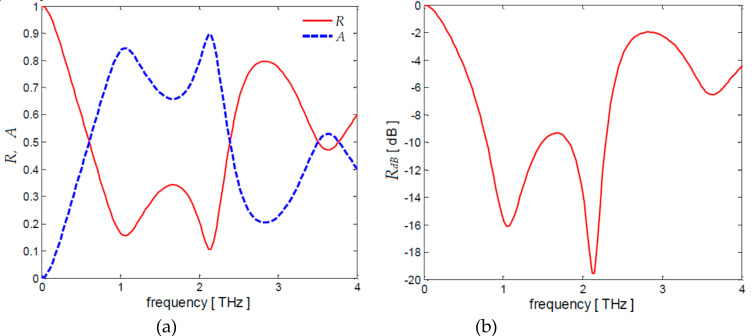
Frequency spectra of coefficients (**a**) *R,T,A* and (**b**) *R_dB_* for *τ* = 0.1 ps, *t_sp_* = 0.035 mm in case of a perfect electric conductor (PEC) mirror layer, i.e., T^*^ = 0.

The previous calculations are carried out considering the laminate *L*_2_ as backside reflector. If the backside reflector is made of a PEC layer, i.e., if T^*^ = 0 and *R_s2_* = 0, it yields *R_s_*_1_ = 125 Ω/sq. The computed frequency spectra of coefficients *R*, *T*, *A* and of *R_dB_* are reported in Figure 6a,b. It is noticed that they are very similar to the ones shown in Figure 5 except for the increased peak value at second resonant frequency.

**Figure 7:**
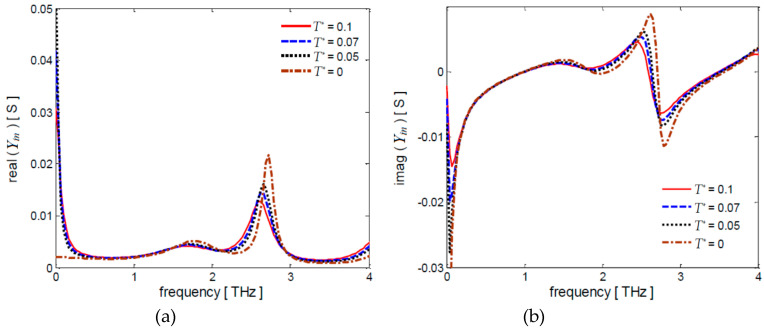
Frequency spectra of the (**a**) real and (**b**) imaginary parts of the input admittance *Y_in_* for τ = 0.1 ps, *t_sp_* = 0.035 mm and different values of T^*^.

For the sake of completeness, the input admittance *Y_in_* of the multilayer screen is computed by using Equation (31) with *τ* = 0.1 ps and *t_sp_* = 0.035 mm. The obtained frequency spectra of the real and imaginary parts of *Y_in_* are shown in Figure 7a,b for different values of T^*^.

At a first resonance frequency of 1.1 THz the real and imaginary parts of *Y_in_* are equal to (1/*η*_0_) = 2.65 ms and zero, respectively, according to the optimal absorbance conditions assumed to obtain the analytical expressions of the sheet conductances.

It is interesting to compare the reflection coefficient computed when a PEC is considered as a backside reflector with the absorbance performance of the adaptive absorber described in [18]. This absorbing screen has a total thickness of 70 μm, it is backed by a PEC plate and it is made firstly of a graphene lossy sheet and of a multilayer graphene-silicon dioxide (SiO_2_) laminate, sandwiched between two PET dielectric layers. A reflection coefficient of -18 dB was obtained at about 1.1 THz when the graphene-SiO_2_ laminate was electrically doped by a 0.02 V/nm electrostatic field bias, giving rise to a laminate-sheet resistance of about 123 Ω/sq [18]. This value is very close to the one resulting from Equation (44) when the laminate *L*_2_ is replaced by a PEC layer.

The design of a feasible absorber requires the choice of doped graphene layers characterized by suitable values of sheet resistances in order to obtain laminates having effective sheet resistances close to the ones reported in Table 1. With this purpose, HNO_3_ chemically doped graphene layers, characterized by a dc conductivity of 8.33 ms and a sheet resistance of 120 Ω/sq [24], should be selected. In fact, values close to *R_s_*_1_ = 135 ms and *R_s_*_2_ = 31 ms corresponding to T^*^ = 0.07 in Table 1 can be obtained using a single doped graphene layer in the first laminate *L*_1_ and four doped graphene layers in the back laminate *L*_2_.

**Figure 8:**
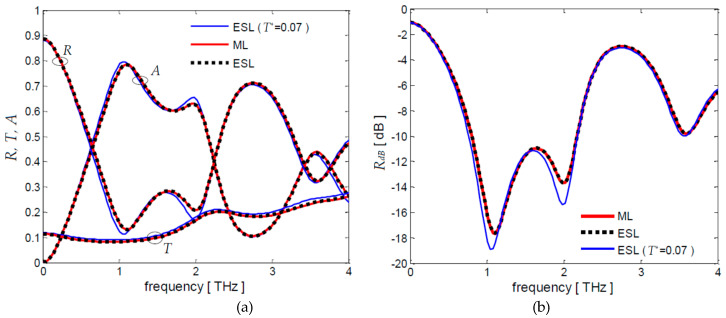
Frequency spectra of coefficients (**a**) *R*, *T*, *A* and (**b**) *R_dB_* for the designed screen obtained using ML- and ESL-short-line models. As a comparison, the (**a**) *R*, *T*, *A* and (**b**) *R_dB_* frequency spectra obtained considering the theoretical sheet resistances obtained for T* = 0.07 (Table 1) are reported.

The coefficients *R*, *T*, *A* and *R_dB_* of the designed absorber are computed assuming *τ* = 0.1 ps and *t*_sp_ = 0.035 mm, as in the previous applications, and using the overall transfer matrix Equation (11) in which each laminate is represented by the multilayer (ML) matrix of Equation (12) or by the ESL-short-line matrix of Equation (26) obtained considering the sheet resistances of the doped graphene layers and the theoretical ones evaluated in the case T* = 0.07 (Table 1). The obtained results are shown in Figure 8a,b.

It can be noticed that the ML- and ESL-short-line spectra are practically coinciding and they are very close to the ones computed by using the theoretical sheet resistance values. Furthermore, the designed absorber has a 1.4 THz bandwidth centered at 1.5 THz with a reflection coefficient below −10 dB.

**Figure 9:**
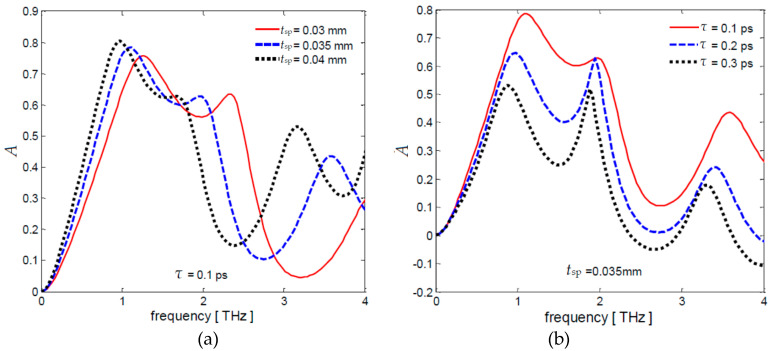
Frequency spectra of absorber coefficient *A* for T^*^ = 0.07 setting (**a**) *τ* = 0.1 ps and (**b**) *t_sp_* = 0.035 mm.

Finally, a sensitivity analysis of the absorption coefficient *A* is carried out for T^*^ = 0.07 with respect to different values of *t*_sp_ and of *τ*, setting respectively *τ* = 0.1 ps or *t*_sp_ = 0.035 mm. The obtained frequency spectra are represented in Figure 9a,b.

The first resonance frequency in Figure 9a is between 0.9 THz and 1.28 THz, depending on the spacer thickness ranging between 0.03 mm and 0.04 mm. The influence of the scattering time on the frequency spectra is noticeable in Figure 9b, showing very different peak values and resonance frequencies for small changes of *τ*. In fact, the frequency dependence of graphene conductivity is more evident as the scattering time increases. These results highlight the importance of an accurate estimation of the scattering time, that is indicative of the graphene quality since it is strictly related to the carrier mobility and chemical potential [21].

## 8. Absorber Transparency

**Figure 10:**
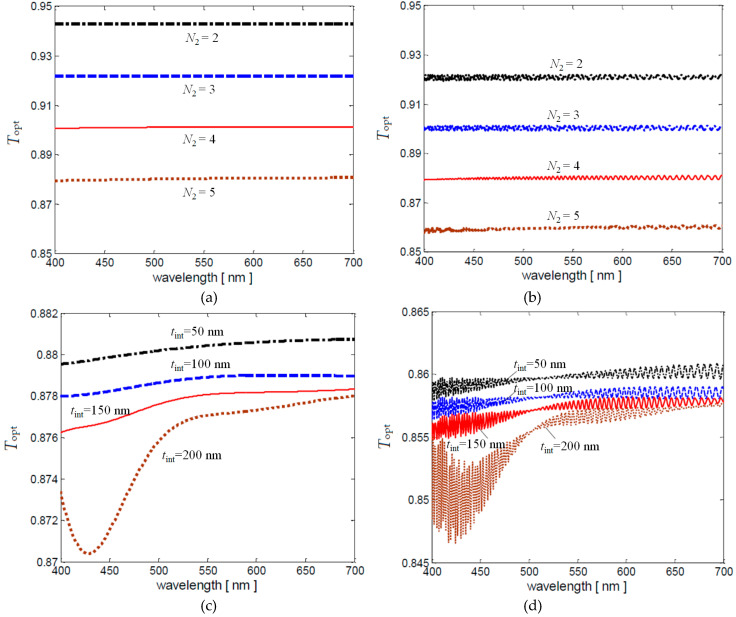
Optical transmittance *T*_opt_ of the (**a**,**c**) back laminate *L*_2_ and (**b**,**d**) of the overall absorbing structure. The spectra are computed in the visible range considering *t*_int_ = 50 mm and *N*_2_ ranges between 2 and 5 (**a**,**b**) or *N*_2_ = 5 and *t*_int_ varying from 50 nm to 200 nm (**c**,**d**).

The optical transmittance *T*_opt_ of the absorber structure sketched in Figure 1 is computed by means of Equation (46), considering the back laminate transfer matrix [*M_L_*_2_] or the overall matrix [*M*], respectively given by Equations (48) and (51). In particular, a sensitivity analysis of *T*_opt_ with respect to the number of graphene layers *N*_2_ of the back laminate *L*_2_ and to the interlayer thickness *t*_int_ is carried out. Figure 10a shows *T*_opt_ spectra of the back laminate having *t*_int_ = 50 nm and *N*_2_ ranging between 2 and 5, while Figure 10b reports *T*_opt_ spectra of the corresponding overall structures, i.e., also considering the matching layer, the laminate *L*_1_ and the spacer. On the other hand, Figure 10c reports *T*_opt_ spectra of the back laminate consisting of five doped graphene layers (*N*_2_ = 5) and interlayer thickness *t*_int_ varying from 50 nm to 200 nm. Similarly, Figure 10d shows *T*_opt_ of the corresponding overall structures, i.e., also considering the matching layer, the laminate *L*_1_ and the spacer.

It can be noticed that *T*_opt_ is always greater than 85%. Moreover, when the PET interlayer thickness is one order of magnitude lower than the considered wavelength, *T*_opt_ can be easily computed by means of the approximated expression Equation (8). As the PET interlayer thickness increases, *T*_opt_ cannot be computed by means of Equation (8), as shown in Figure 10c. In fact, mutual reflections inside the interlayer are no more than negligible.

Finally, from Figure 10b it results that *T*_opt_ of the designed screen (with *N*_2_ = 4) is around 88% in all the visible ranges.

## 9. Conclusions

The proposed absorber structure consists of a front matching PET layer followed by a graphene/PET laminate, a PET spacer and a back graphene/PET laminate. The matching layer and the spacer have one-quarter-wavelength thickness. The structure is extremely simple since it does not include complex frequency-selective surfaces or metamaterials. Moreover, the designed absorbers have a total thickness of 70 µm and they are transparent and metal free, since no metal is used, also for the backside reflector.

The absorption performances are evaluated through numerical calculations in the low-terahertz frequency range and both graphene conductivity and PET permittivity are considered as frequency-dependent quantities. A homogenization procedure is applied to represent the periodic graphene-PET structures as equivalent short-line single layers.

The optimum sheet resistances of the laminates are obtained analytically as functions of the interlayer relative permittivity and of the target transmission coefficient at the first resonance frequency. It is worth noticing that the analytical expressions of the optimum laminate-sheet resistances can be applied to both low-gigahertz or low-terahertz frequency ranges. Then, HNO_3_ chemically doped graphene layers are considered in order to design feasible absorbing screens with laminate-sheet resistances comparable with the theoretical ones. The computed frequency spectra of the absorption, reflection and transmission coefficients in the frequency range up to 4 THz prove the good performance of the designed absorbers and the utility of the proposed design method. In particular, the proposed screen has a 1.4 THz bandwidth centered at 1.5 THz with reflection coefficients below - 10 dB and an absorption coefficient with peak values around 0.8 at the first resonant frequency of 1.1 THz. Then, a sensitivity analysis is carried out to highlight the strong influence of the estimated scattering time on the screen performances.

Finally, the optical transmittance of the proposed absorber is computed by means of the optical matrix theory and we conclude that such transmittance is greater than 88% in all the visible ranges.

## Figures and Tables

**Table 1:** Sheet Resistances of Graphene-PET Laminates.

T*	*R_s_*_1_ [Ω/sq]	*R_s_*_2_ [Ω/sq]
0.10	141	47
0.07	135	31
0.05	133	21
